# An External CAM Therapy (Tian Jiu) versus Placebo in Treatment of Allergic Rhinitis: A Pilot Single-Blinded, Three-Arm, Randomized Controlled Study

**DOI:** 10.1155/2019/6369754

**Published:** 2019-04-14

**Authors:** Liang Dai, Linda L. D. Zhong, Wai Kun, Wai Ching Lam, Zhen Yang, Tao Huang, Huaixue Mu, Zhao-xiang Bian

**Affiliations:** Hong Kong Chinese Medicine Clinical Study Centre, School of Chinese Medicine, Hong Kong Baptist University, Hong Kong, China

## Abstract

Allergic rhinitis (AR) is one of the common allergic diseases in clinical practice and significantly impairs the quality of life (QoL) of patients. The conventional treatments are not satisfactory because of various reasons. Tian Jiu (TJ) therapy is a characteristic external intervention of complementary and alternative medicine (CAM) and has been widely utilized in the management of AR. However, the evidences resulted from current studies were generally poor due to high risk of bias. Therefore, we conducted this rigorous designed, single-blinded, three-arm, randomized controlled study to evaluate the efficacy and safety of TJ therapy on AR. Totally 138 AR patients were enrolled. The TJ group and placebo group received 4-week treatment with either TJ or placebo patches for 2 hours each time applied to Dazhui (GV 14), bilateral Feishu (UB 13), and bilateral Shenshu (UB 23) one session per week and then underwent a 4-week follow-up. The waitlist group received no treatment during the corresponding treatment period, but would be given compensatory TJ treatment in the next 4 weeks. The primary outcome was the change of the Total Nasal Symptom Score (TNSS) after treatment. The secondary outcomes included the changes of Rhinoconjunctivitis Quality of Life Questionnaire (RQLQ) and rescue medication score (RMS). After treatment, the total TNSS in TJ group was significantly reduced compared with baseline, but showed no statistical meaning compared with placebo. Among the four domains of TNSS, the change of nasal obstruction exhibited statistical difference compared with placebo group. The total RQLQ score in TJ group was significantly reduced compared with both placebo and waitlist groups. The needs of rescue medications were no distinct difference between two groups. In summary, this study showed potential effectiveness of TJ therapy in improving nasal obstruction symptoms and QoL of AR patients.

## 1. Introduction

Allergic rhinitis (AR), affecting 10% to 20% of the population globally, is one of the common allergic diseases in clinical practice [1]. In China, based on the epidemiological surveys conducted in major cities, the prevalence of self-reported AR had increased from 11.1% to 17.6% in a 6-year period (2005 to 2011) [2, 3]. Although AR is not a life-threatening disease, the classical nasal and ocular symptoms, including nasal obstruction and pruritus, eyelid edema, and asthenia, significantly impair the quality of life (QoL) of patients [4–7]. Routine symptomatic pharmacotherapies contain H_1_-antihistamines, intranasal glucocorticoids, and leukotriene-receptor antagonists [8], while it has been reported that only one-third of adults experienced satisfactory relief after treatment [9]. For the non-responders, immunotherapy would be considered in next step. However, the optimal use, appropriate initiation timing, and proper duration of immunotherapy are still difficult to determine [8]. Therefore, many patients would seek help from complementary and alternative medicine (CAM), especially Traditional Chinese Medicine (TCM) in East Asia [10–12].

Tian Jiu (TJ), also known as “Chinese Medicine moxibustion” or “acupoint Chinese Medicine patching”, is a typical external intervention of TCM and has been utilized in multiple respiratory diseases in China for a long history [13]. During TJ treatment, pre-prescribed acupoints were attached by Chinese Medicine patches. On the foundation of TCM theory, this intervention could regulate the functions of meridians and viscera and thereby invigorate physical institution so as to prevent and treat diseases [14]. In addition, the abnormal body institution is considered to be the internal reason of AR attack according to TCM concept; thus it is reasonable to apply TJ in management of AR [15]. The specific mechanisms of the effect of TJ therapy are still not clean, but multiple systems were reported to be involved [16]. Animal researches reported that the TJ could reduce the level of peripheral blood eosinophils and serum immunoglobulin E (IgE), inhibit mast cell degranulation, decrease the production of inflammatory cytokines, and suppress lymphocyte immune response [17–20]. Previous meta-analysis has reported the efficacy and safety of AR in TJ treatment. TJ alone or in combination with Western medicine showed better effectiveness than placebo or Western medicine, respectively. However, the conclusion resulted from trials with high risk bias and should be interpreted with caution [21].

Therefore, for the purpose of further evaluating the effectiveness of TJ therapy in the management of AR, we conducted this rigorous designed trial by evaluating the generally accepted symptoms scales, Total Nasal Symptom Score (TNSS), and Rhinoconjunctivitis Quality of Life Questionnaire (RQLQ). The hypothesis was that TJ group would significantly alleviate symptoms and improve QoL of AR patients when compared with placebo and waitlist groups.

## 2. Materials and Methods

### 2.1. Study Design

It was a prospective, randomized, single-blinded, controlled clinical study. A total of 138 participants were included from the public by advertisements on website and local newspaper. After a one-week run-in period, qualified participants were randomly allocated to TJ arm, placebo arm, and waitlist arm. TJ group and placebo group received either Chinese Medicine patches or placebo patches one session per week continuously for 4 weeks, respectively. After treatment period, another 4-week follow-up was set to evaluate the sustained effects. The waitlist group was given no intervention during the first 4 weeks, but, after the waiting period, participants in waitlist group were given compensatory TJ treatment for 4 weeks. Based on this design, the effect of TJ and placebo could be evaluated simultaneously. All groups were evaluated identically during the whole study. The total study period was nine weeks.

The study protocol was approved by the Hong Kong Baptist University Ethics Committee on the Use of Human Subjects for Teaching and Research (Approval no. HASC/13-14/0241). The protocol has been registered with the identifier NCT02470845 (17 May 2015) at ClinicalTrials.gov and published in* Trials *[22]. Every included patient signed the Informed Consent voluntarily. The checklists for items in CONSORT 2010 and STRICTOM 2010 were given in* Supplement *1 and* Supplement *2, respectively [23, 24]. The research was supported by the Marcoda Co. Ltd., which had no role in the protocol design, study implementation, data collection, and manuscript preparation.

### 2.2. Participants

*Setting.* The study was conducted in the research and clinical centers, School of Chinese Medicine, Hong Kong Baptist University (HKBU). 

*Inclusion Criteria.* Patients who met all following criteria were defined as qualified participants: (1) both genders aged at least 18 years old; (2) having intermittent or persistent AR; (3) severity beyond mild (TNSS ≥8). The diagnostic standard for AR was based on ARIA criteria: typical history of allergic symptoms plus diagnostic tests [20]. The typical symptoms include rhinorrhoea, sneezing, nasal obstruction, and pruritus. Diagnostic tests, such as skin prick test, radio allergo sorbent test (RAST), and nasal provocation, need to demonstrate allergen-specific IgE in the skin or the blood.

*Exclusion Criteria*. Patients who met any of the following criteria were excluded: (1) concomitant with allergic asthma and moderate to severe atopic dermatitis; (2) concomitant with any autoimmune diseases; (3) concomitant with severe chronic inflammatory diseases; (4) history of anaphylactic reactions; (5) hypersensitivity to cetirizine or related drugs; (6) application of specific immunotherapy during the past 3 years or planned in the next 2 years; (7) pregnancy or lactation.

### 2.3. Interventions

*Medications*. All included participants were informed to stop taking related medications, and the application of emergency medications was only allowed when the AR symptoms were uncontrollable. Patients in the TJ group and placebo group were treated with either Chinese Medicine patches or placebo patches on five identical acupoints on the back, respectively. The patches were bought from licensed medical device supplier. The ingredients of the Chinese Medicine patch contained Bai Jie Zi (*Sinapis semen*), Yan Hu Suo (*Corydulis rhizoma*), Zhi Gan Sui (*Kansui radix*), Xi Xin (*Asari radix et rhizama*), and synthesized Artificial She Xiang (*Moschus artifactus*). The composition was modified on the foundation of previous systematic review, preliminary survey among experienced TCM practitioners, and routine TJ formula used in HKBU clinics for more than ten years [21, 25, 26]. The composition and the action of each herb in the TJ Chinese Medicine patches were listed in Table 1.

In order to make high quality Chinese Medicine patch, the contents of crude medicinal materials were determined by high-performance liquid chromatography (HPLC). This method was well validated in terms of linearity, precision, accuracy, recovery, and stability. All calibration curves had good linearity and were shown in* Supplement *3. The precision, accuracy, recovery, and stability were satisfactory. The established HPLC method was successfully applied to the simultaneous determination of euphadienol, asarinin, sinapine thiocyanate, and tetrahydropalmatine. Representative chromatograms of the standard solution and samples were shown in* Supplement *4. All the contents were summarized in* Supplement *5.

The first four herbs were grounded and mixed into a powder, with the ratio of 20% Bai Jie Zi, 25% Yan Hu Suo, 15% Zhi Gan Sui, and 40% Xi Xin. Then the fresh ginger juice was added into the mixed powder according to the ratio of 25mL to 20g. Afterwards, the mixture was processed into 1cm^2^ round patches weighing 2g each. Finally, 0.02g Artificial She Xiang was put on top of each patch. The placebo patch was made of flour and edible pigments. The substances will be mixed with water and made into similar 1cm^2^ round patches weighing 2g to achieve the similarity of the color and shape with Chinese Medicine patches.

*Acupoints. *Based on previous systematic review, preliminary survey among experienced TCM practitioners, and routine TJ regimen applied in HKBU clinics, Dazhui (GV 14), bilateral Feishu (UB 13), and bilateral Shenshu (UB 23), five acupoints in total were selected [21, 25, 26]. Participants in TJ group and placebo group received a 4-week continuous treatment with either Chinese Medicine patches or placebo patches applied to each acupoint weekly, and then underwent a 4-week post-treatment follow-up. Patients in the waitlist group were not allowed getting any treatment during the same period but would be given compensatory TJ treatment in next 4 weeks. A 2cm *∗* 2cm piece of hypoallergenic tape was utilized to paste the patches to each acupoint. The retention time was 2 hours. All operations were completed by our TCM practitioners who should be registered TCM practitioners with no less than 5 years of TCM undergraduate education and more than 5 years of clinical experience. The patching time could be properly shortened if the patient could not tolerate the stimulation or appeared allergic reactions. Names and details of acupoints were presented in Table 2.

### 2.4. Outcomes

The primary outcome was set to be the difference in the weekly average of the TNSS documented in patients' diaries aftertreatment. Four nasal symptoms, namely, rhinorrhea, nasal itching, nasal obstruction, and sneezing, compose the TNSS. A five-point scale from 0 to 4 (0 = no symptoms; 1 = mild; 2 = moderate; 3 = severe; 4 = very severe) was applied to evaluate the severity of the symptoms. The TNSS was the summation of four individual symptom scores, ranging from 0 (no symptom) to 16 (maximum symptom intensity). This scoring system has been generally utilized in efficacy evaluation of AR treatment [27, 28].

The secondary outcomes included the assessment of QoL and the rescue medication needs. The QoL was evaluated by using RQLQ, which has 28 questions in seven domains (sleep, non-rhinoconjunctivitis symptoms, practical problems, nasal symptoms, ocular symptoms, activity limitations, and emotional function) ranging from 0 (no impairment) to 6 (maximum impairment) [29]. The rescue medication needs were assessed by using rescue medication score (RMS). RMS was calculated daily on a 4-point scale (0 = no rhinitis medicine; 1 = cetirizine, 10 mg/d, or equivalent; 2 = cetirizine, 20 mg/d, or equivalent; 3 = systemic or topical corticosteroids for nose or lung) (daily range; 0 to 3; weekly range: 0 to 21). When more than one rescue medication was applied in one day, only the highest level medication was documented [30].

Adverse events were collected throughout the study, on the foundation of participant-reports and practitioners' consultation. If serious adverse events occurred, the treatment would be suspended immediately, and whether the trial should be suspended would be decided after further assessment.

### 2.5. Randomization Assignment

This study was a randomized controlled trial. Qualified participants were randomly distributed to TJ arm, placebo arm or waitlist arm according to the ratio of 1:1:1. Random Allocation Software (version 1.0.0, Isfahan, Iran) was utilized to generate a randomization scheme. The principal investigator (PI) generated the random allocation sequence and was not allowed to inform other researchers. After the run-in stage, a research assistant (RA) assigned the treatments based on the codes, which were preserved in opaque sealed envelopes with consecutive randomization numbers. Emergency unblinding only happened when the intervention details were necessary for the patient's future management. Such arrangement would ensure the clinical evaluator and subjects to be blind to the allocation.

### 2.6. Sample Size Calculation

Sample size was estimated according to the primary outcome. As this was a pilot clinical trial, we assumed the improvement rate of waitlist group would be 0. Afterwards, we supposed that there would be 30% improvement of TNSS in TJ group and 15% in placebo group. Considering a power of 80%, and *α* value of 2.5 % (two-tailed), the sample size will be calculated using the following formula:

(1) $$\begin{array}{c} n=\frac{2\lambda }{{\left(2\sin^{-1}\sqrt{Pt}-2\sin^{-1}\sqrt{Pw}\right)}^{2}} \end{array}$$

The *λ* was the cut-off value. Pt is 30% and Pw is 0 off value. 38 subjects in each were needed for the detection of significance [31]. Considering of a 20 % dropout, the sample size was increased to 138 in total, with 46 participants in each arm.

### 2.7. Data Processing and Analysis

All efficacy and safety analyses were conducted based on intention-to-treat (ITT) principle. Missing values were imputed by the last-observation-carried-forward method. The statistical analysis was performed using the Statistical Packages of Social Sciences (SPSS) for Windows version 21.0. The statistical significance was defined as two-sided P-value of <0.05. Baseline characteristics were reported as mean (standard deviation (SD)) or frequency. Baseline differences among the groups were evaluated with the application of analysis of variance (ANOVA) for normally distributed continuous variables and non-parametric Kruskal-Wallis* H* test for non-normally distributed variables. For categorical variables, chi-squared test or Fisher's exact test was applied. Comparisons between groups were conducted by using an analysis of covariance (ANCOVA) with baseline as covariate. All items and subscales were compared between groups for each 4-week using ANCOVA, with treatment group as a factor in the model and baseline as the covariate. The differences from baseline to the end of treatment in scores were tested with repeated measure ANOVA. Within group differences were evaluated with ANOVA and post hoc comparison for normally distributed data and Wilcoxon signed-rank test for non-normally distributed data.

### 2.8. Data Collection and Handling of Withdrawal and Dropout

This was a 9-week clinical study, in which participants required to receive TJ treatment for 4 weeks, attend five assessment visits, finish a series of questionnaires, and avoid using other symptomatic treatments. All collected information was input into electronic file by a RA. Simultaneously, all files were stored in numerical order and kept in storage for 5 years. In order to ensure the maximum compliance, before the treatment, a consent process was conducted to inform the study arrangements, potential adverse events, and participants' responsibilities to all patients. Additionally, a RA would contact the participant to re-confirm the schedule before every visit. When a participant showed signs of withdrawl, we would try to explore the potential causes and seek the suitable solutions to ensure the compliance.

## 3. Results

### 3.1. Baseline Characteristics

**Figure 1:**
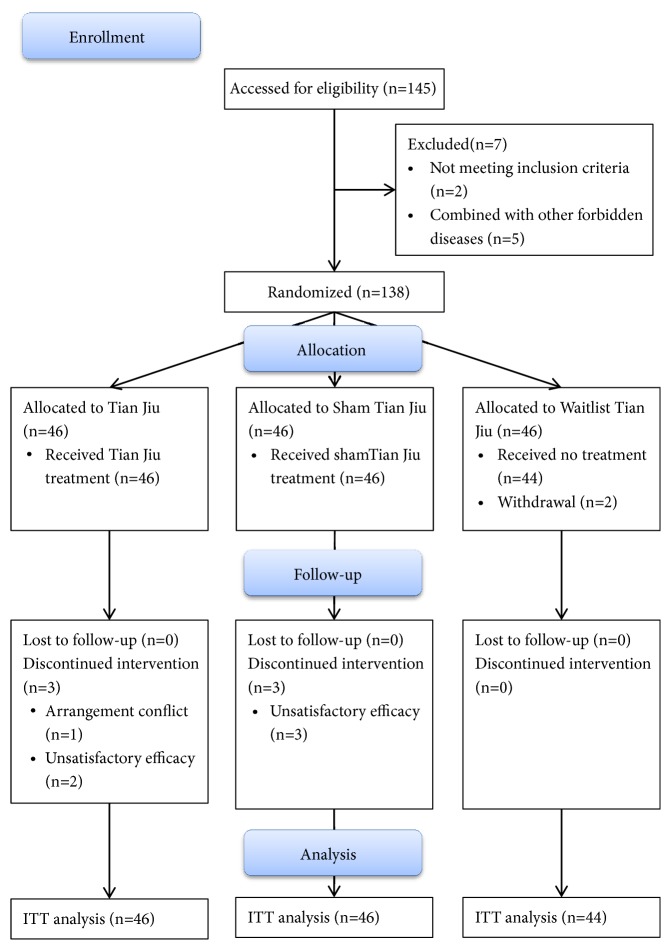
Participants flow diagram.

From May 2016 to October 2016, 245 participants were screening and 138 participants were randomized. The flow diagram of patients was showed in Figure 1. The baseline characteristics of three groups were presented in Table 3. The distributions of the demographic and clinical characteristics were well balanced and homogeneous (*P*>0.05).

### 3.2. TNSS Change

**Figure 2:**
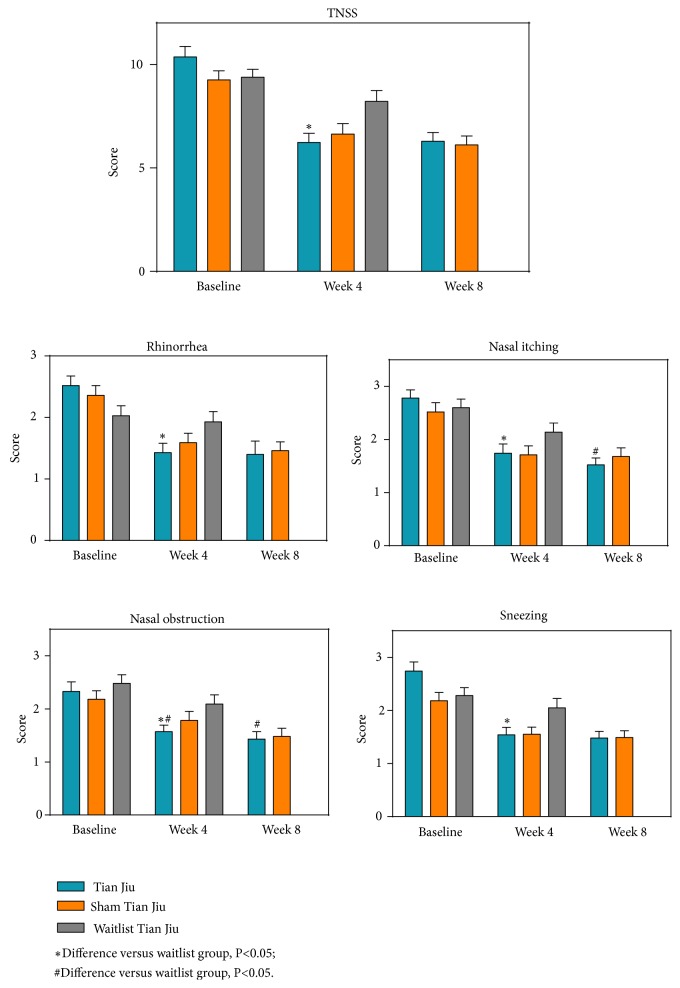
Comparison of TNSS between three groups.

After 4-week treatment, the total TNSS in TJ group was significantly decreased from 10.37 (SD: 3.40) to 6.24 (SD: 2.94). The change had statistical difference compared with waitlist group (P=0.003), while showing no statistical difference compared with placebo group (9.25 (SD: 3.05) to 6.63 (SD: 3.49), P=0.466) (Figure 2). Among the four elements, the change of nasal obstruction score had statistical difference compared with the placebo group after 4-week treatment (from 2.33 (SD: 1.22) to 1.57 (SD: 0.83) compared with from 2.18 (SD: 1.08) to 1.78 (SD: 1.17), P=0.046), and the effect on nasal obstruction remained in the 4-week follow-up (from 2.33 (SD: 1.22) to 1.43 (SD: 0.96) compared with from 2.18 (SD: 1.08) to 1.48 (SD: 1.05), P=0.044) (Figure 2). Additionally, the change of nasal itching score showed no statistical difference compared with the placebo group (from 2.78 (SD: 1.06) to 1.74 (SD: 1.20) compared with from 2.52 (SD: 1.17) to 1.71 (SD: 1.15), P=0.073); however, the effect revealed in the 4-week follow-up (from 2.78 (SD: 1.06) to 1.52 (SD: 0.90) compared with from 2.52 (SD: 1.17) to 1.68 (SD: 1.09), P=0.021) (Figure 2). Besides, the score of each symptom in TJ group was significantly reduced compared with waitlist group (Figure 2). The detailed changes of TNSS were shown in Table 4.

### 3.3. RQLQ Change

After 4-week treatment, the total RQLQ score in TJ group reduced from 2.32 (SD: 1.35) to 1.82 (SD: 1.19), while the placebo group and waitlist group only showed the trend of fluctuation (1.91 (SD: 1.48) to 1.95 (SD: 1.49) in placebo group and 1.49 (SD: 1.13) to 1.49 (SD: 1.13) in waitlist group). The difference compared with either group had statistical significance (P=0.006 and P=0.008, respectively). Moreover, the TJ group remained the slight decreasing trend in the 4-week follow-up (from 1.82 (SD: 1.19) to 1.80 (SD: 1.19), and the change had statistical significance compared with placebo group (P=0.009). With regard to the seven domains in RQLQ, after 4-week treatment, the TJ therapy benefited non-rhinoconjunctivitis symptoms, practical problems, nasal symptoms and activity limitations compared with non-intervention (waitlist group), and it showed superior efficacy than the placebo in sleep, non-rhinoconjunctivitis symptoms, nasal symptoms, and activity limitations. Then after 4-week follow-up, the TJ therapy still showed advantages in sleep, non-rhinoconjunctivitis symptoms, practical problems and nasal symptoms compared with placebo. The detailed changes of RQLQ were shown in Table 5.

### 3.4. Safety Assessments

There were no serious adverse events that happened during the whole treatment. The common adverse events included flush, pruritus, blister, and pigmentation, occurring in 17, 23, 3, and 36 person-times among TJ group, and 3, 7, 1, and 4 person-times among placebo group, respectively. These adverse events were generally tolerated and disappeared quickly after removing the patches. For the rescue medication needs, the RMS could not be calculated because only few participants took emergency medications. To be specific, during the 4-week treatment, 2 person-times used medication equivalent to cetirizine 10 mg/d, and 1 person-time used systemic or topical corticosteroids for nose or lung in TJ group. The frequency and categories of applied rescue medications were exactly the same in placebo group. Then during the 4-week follow-up, the frequencies of using medication equivalent to cetirizine 10 mg/d were 3 and 2 in TJ group and placebo group, respectively, which also showed no distinct difference.

## 4. Discussion

TJ therapy is a typical external intervention of TCM and has been still widely utilized in clinical practice in China. Our results demonstrated certain effect of TJ therapy in improving nasal obstruction and quality of life of AR patients during treatment period, despite the total TNSS of TJ group did not show significant difference compared with placebo. In addition, the treatment effect could remain after the TJ therapy withdrawal. The study presented relatively consistent results of previous studies [21, 25, 26].

TJ therapy has a long history in clinical practice. The ancient classic “Zhang Shi Yi Tong” in Qing Dynasty firstly documented using Bai Jie Zi patching to treat cold wheezing. According previous statistics, AR ranked the third among the most commonly diseases treated by TJ therapy [11]. Comparing with other TCM external interventions, TJ therapy has its own advantages. For example, TJ therapy is easier to be self-handled. Unlike acupuncture and moxibustion, TJ could be completed by patients themselves if detailed patching locations are given. In addition, the adverse events are slight. As shown in our results, the major adverse events would disappear quickly without any special managements. Relatively, the risk of adverse events of other interventions such as acupuncture was higher, including bleeding and stabbing pain [32].

The basic treatment principles against AR include immune-regulation and anti-inflammation. Previous studies have also illustrated TJ's effects on these two aspects. The enhanced IgE level is a vital factor in AR pathogenesis. It could trigger the release of inflammatory mediators, such as histamine and leukotrienes, which would induce arteriolar dilation, vascular permeability elevation, itching, rhinorrhea (runny nose), mucous secretion, and smooth muscle contraction [33, 34]. Previous researches have found that TJ treatment could significantly decrease the serum IgE [35–38]. In addition, studies have also showed than TJ therapy could regulate the level of certain cytokines, for instance, decreasing interleukin-4 (IL-4), interleukin-5 (IL-5), and tumor necrosis factor-*α* (TNF-*α*) that may reduce the production of IgE from upstream [38]. Moreover, it has also been proven that TJ treatment could downregulate the level of eosinophil cationic protein (ECP), which meant TJ also had certain anti-inflammation effect [35, 37]. Our study did not involve any relevant blood tests to evaluate the immune state and inflammation level of participants. This should be filled up in future studies.

The treatment effect of TJ therapy could be affected by multiple factors, including treatment timing, frequency, retention time, and body response [39]. Our study conducted the intervention under the most basic conditions, namely, normal days, routine treatment sessions (4 times) and patching time (2 hours), and no strict body response requirements, therefore to evaluate the fundamental efficacy of TJ therapy. Further research is worth being conducted. Firstly, the treatment timing could be moved to Sanfu days (the hottest days of the year). Recent study has reported that TJ therapy in Sanfu days had better long-term efficacy than normal TJ therapy, which indicated that the therapeutic effect of Sanfu TJ therapy may remain longer [36]. Secondly, increasing treatment sessions and enhancing patching time could be considered. Xu et al. has found that there was a positive correlation between the efficacy of TJ therapy and treatment years. The longer the patients applied TJ therapy, the more stable the efficacy obtained [40]. In addition, referring to the published systematic review, most studies set the patching time as 4-6 hours, while our study only set 2 hours [21]. The extended retention time increased the duration of local irritations and then may contribute the efficacy. Besides, there has also been reported that blistering TJ had better efficacy than non-blistering TJ [41]. Nevertheless, considering that blistering TJ is a traumatic external intervention, the application may not be smooth.

Our study has several limitations. Although the pasting procedure of TJ patches was completed by our practitioners, the peeling was finished by participants themselves. Some participants may not tolerate the stimulation, or remember the time incorrectly. The patching time could be shortened or extended, then led to potential bias. Besides, the follow-up period was only 4 weeks, which was not enough to assess the long-term effect. The future study should consider at least 6-month follow-up period or even longer to evaluate the persistent effect. Thirdly, the sample size of this pilot study was calculated based on primary outcome. We assumed the waitlist group would have no improvement. However, after the analysis, surprisingly we found the TNSS in waitlist group had 12.4% decline. This effect may have resulted from the environment and/or psychological impact. This ignored effect did not involve in the sample size calculation and then may lead to the underestimation of needed participants.

## 5. Conclusion

In summary, this randomized, single-blinded, controlled trial served primary evidence of the efficacy and safety of TJ therapy on AR in Hong Kong. This pilot study provided a fundamental TJ protocol for future research. Through adjusting treatment timing, frequency, retention time, and even body response settings, it has the potential to develop into an optimal therapeutic method for future application.

## Figures and Tables

**Table 1:** Composition and action of Tian Jiu treatment formula.

Ingredients	%	Action
*Sinapis Semen*	8.79	TCM: (1) To warm the lung and sweep phlegm, (2) To disinhibit qi, (3) To dissipate binds and unblock collaterals to relieve pain
		Pharmaceutical study: (1) Antibacterial effect, (2) Skin stimulating effect, (3) Effect on gastric secretion, (4) Emetic effect, (5) Regulation of blood pressure, (6) Anti-lipid peroxidation, (7) Expectorant effect

*Corydulis Rhizoma*	11.01	TCM: (1) To activate blood, (2) To move qi, (3) To relieve pain
		Pharmaceutical study: (1) Effect on the CNS (analgesic effect, hypnotic effect, sedative and tranquilizing effects), (2) Muscular relaxant effect, (3) Cardiovascular effect, (4) Effect on gastric secretion and on urination, (5) Effect on pituitary-adrenocortical function

*Kansui Radix*	6.56	TCM: (1) To expel retained fluid by purgation, (2) To disperse swelling and dissipate binds
		Pharmaceutical study: (1) Purgative effect

*Asari Radix et rhizama*	17.69	TCM: (1) To dispel wind and dissipate cold, (2) to dispel wind and relieve pain, (3) To open the orifices, (4) To warm the lung and resolve fluid retention
		Pharmaceutical study: (1) Sedative and analgesic effects, (2) Antipyretic and anti-inflammatory effects, (3) Respiratory effect, (4) Cardiovascular effect, (5) Anti-histaminic and anti-allergic effects

Synthesized *Moschus Artifactus*	0.45	TCM: (1) To induce resuscitation and restore consciousness, (2) To promote blood circulation to remove obstruction, (3) To alleviate pain
		Pharmaceutical study: (1) Effects on the CNS, (2) cardiovascular effects, (3) Anti-inflammatory effect, (4) Effects on the uterus

*Zingiberis Rhizoma Recens (fresh ginger juice)*	50	TCM: (1) To release the exterior and dissipate cold, (2) To warm the middle energizer to check vomiting, (3) To resolve phlegm and suppress cough, (4) To reduce toxin of fishery product
		Pharmaceutical study: (1) Effects on the digestive system, (2) Antiemetic effect, (3) Effect on the CNS

**Table 2:** Acupuncture points used in the study.

Name	Areas	Special Qualification	Effects of Stimulation	Indications
Dazhui (GV 14)	In a depression below the processus spinosus of the 7^th^ cervical and above the 1^st^ thoracic vertebrae.	A copulo-conventory by which the leading sinartery communicates with all yang conduits.	Stabilising and regulating the qi of centre; strengthening the hepatic and pulmonal orb, keeping the nasal open.	Cough and asthma

Feishu (UB 13)	1.5 PI laterally of the processus spinosus of the 3^rd^ thoracic vertebrae	Dorsal inductor (inductorium dorsale) for the pulmonal orb.	Stabilising and harmonizing the pulmonal and the renal orb; regulating the qi; strengthening the yin.	Cough with asthma, common cold, nasal congestion

Shenshu (UB 23)	1.5 PI laterally of the 2^nd^ lumbar vertebrae	Dorsal inductor of the renal orb.	Strengthening and harmonizing the renal orb; improving hearing and clearing vision.	Cough, asthma, asthenic breathing

**Table 3:** Baseline characteristics.

Characteristic	Tian Jiu (N=46)	Sham Tian Jiu (N=46)	Waitlist Tian Jiu (N=44)	P Value
Sex, *n (*%)				
Female	23 (50.0)	25 (54.3)	20(47.8)	0.502
Male	23 (50.0)	21 (45.7)	24 (52.2)	
Age, Mean (SD), y	45.6 (15.1)	40.4 (15.6)	38.6(14.4)	0.807
Weight, Mean (SD), kg	64.9 (19.7)	64.8 (21.3)	59.8 (15.0)	0.878
Height, Mean (SD), cm	165.2 (11.3)	161.6 (23.6)	160.1 (15.9)	0.400
BMI, Mean (SD), kg/m2	22.5 (3.2)	23.1 (3.0)	22.2 (3.9)	0.704
Duration of AR, y	17.1 (11.8)	15.0 (8.1)	14.6 (10.1)	0.280
TNSS, Mean (SD)	10.37(3.4)	9.25(3.05)	9.38(2.57)	0.664
Rhinorrhea	2.52(1.04)	2.36(1.08)	2.03(1.07)	0.118
Nasal itching	2.78(1.06)	2.52(1.17)	2.6(1.06)	0.357
Nasal obstruction	2.33(1.22)	2.18(1.08)	2.48(1.09)	0.803
Sneezing	2.74(1.17)	2.18(1.06)	2.28(0.99)	0.504
RQLQ, Mean(SD)	2.32(1.35)	1.91(1.48)	1.49(1.13)	0.349
Sleep	2.48(1.46)	2.23(1.81)	1.92(1.59)	0.491
Non-rhinoconjunctivitis symptoms	2.44(1.27)	2.05(1.55)	1.65(1.11)	0.103
Practical problems	2.83(1.27)	2.48(1.49)	1.96(1.26)	0.413
Nasal symptoms	2.77(1.32)	2.41(1.33)	1.81(0.97)	0.317
Ocular symptoms	1.89(1.59)	1.36(1.31)	0.99(1.07)	0.771
Activity limitations	2.06(1.13)	1.62(1.29)	1.19(0.90)	0.096
Emotional function	1.82(1.38)	1.23(1.60)	0.91(1.00)	0.646

**Table 4:** The TNSS in different visits.

	Tian Jiu (N=46)	Sham Tian Jiu (N=46)	Tian Jiu vs Sham Tian Jiu P-value*∗*	Waitlist Tian Jiu (N=44)	Tian Jiu vs Waitlist Tian Jiu P-value*∗*
TNSS					

Baseline	10.37(3.40)	9.25(3.05)	-	9.38(2.57)	-
Week 4	6.24(2.94)	6.63(3.49)	0.466	8.21(3.56)	0.003
Week 8	6.28(2.95)	6.11(2.95)	0.582	-	-

*Rhinorrhea *					

Baseline	2.52(1.04)	2.36(1.08)	-	2.03(1.07)	-
Week 4	1.43(1.03)	1.59(1.05)	0.162	1.93(1.11)	0.017
Week 8	1.40(1.01)	1.46(0.98)	0.115	-	-

*Nasal itching*					

Baseline	2.78(1.06)	2.52(1.17)	-	2.6(1.06)	-
Week 4	1.74(1.20)	1.71(1.15)	0.073	2.14(1.13)	0.016
Week 8	1.52(0.90)	1.68(1.09)	0.021	-	-

*Nasal obstruction*					

Baseline	2.33(1.22)	2.18(1.08)	-	2.48(1.09)	-
Week 4	1.57(0.83)	1.78(1.17)	0.046	2.09(1.16)	0.011
Week 8	1.43(0.96)	1.48(1.05)	0.044	-	-

*Sneezing*					

Baseline	2.74(1.17)	2.18(1.06)	-	2.28(0.99)	-
Week 4	1.54(0.96)	1.55(0.91)	0.804	2.05(1.16)	0.001
Week 8	1.48(0.86)	1.49(0.86)	0.062	-	-

For each variable, the values are expressed as the mean (SD).

*∗*Comparison based on the change of score.

**Table 5:** The RQLQ in different visits.

	Tian Jiu (N=46)	Sham Tian Jiu (N=46)	Tian Jiu vs Sham Tian Jiu P-value*∗*	Waitlist Tian Jiu (N=44)	Tian Jiu vs Waitlist Tian Jiu P-value*∗*
RQLQ					

Baseline	2.32(1.35)	1.91(1.48)		1.49(1.13)	
Week 4	1.82(1.19)	1.95(1.49)	0.006	1.49(1.13)	0.008
Week 8	1.80(1.19)	1.92(1.48)	0.009	-	-

*Sleep*					

Baseline	2.48(1.46)	2.23(1.81)		1.92(1.59)	
Week 4	1.76(1.21)	2.25(1.79)	0.038	1.91(1.59)	0.057
Week 8	1.77(1.21)	2.19(1.79)	0.027	-	-

*Non-rhinoconjunctivitis symptoms*					

Baseline	2.44(1.27)	2.05(1.55)		1.65(1.11)	
Week 4	1.90(1.24)	2.11(1.57)	0.016	1.64(1.11)	0.014
Week 8	1.88(1.22)	2.07(1.55)	0.034	-	-

*Practical problems*					

Baseline	2.83(1.27)	2.48(1.49)		1.96(1.26)	
Week 4	2.40(1.32)	2.56(1.46)	0.609	1.99(1.26)	0.018
Week 8	2.37(1.33)	2.53(1.48)	0.046	-	-

*Nasal symptoms*					

Baseline	2.77(1.32)	2.41(1.33)		1.81(0.97)	
Week 4	2.16(1.12)	2.51(1.32)	0.014	1.83(0.97)	0.018
Week 8	2.14(1.12)	2.49(1.34)	0.046	-	-

*Ocular symptoms*					

Baseline	1.89(1.59)	1.36(1.31)		0.99(1.07)	
Week 4	1.32(1.09)	1.3(1.30)	0.394	0.98(1.08)	0.370
Week 8	1.32(1.08)	1.3(1.30)	0.252	-	-

*Activity limitations*					

Baseline	2.06(1.13)	1.62(1.29)		1.19(0.90)	
Week 4	1.66(1.04)	1.66(1.36)	0.031	1.18(0.89)	0.030
Week 8	1.64(1.02)	1.59(1.29)	0.052	-	-

*Emotional function*					

Baseline	1.82(1.38)	1.23(1.60)		0.91(1.00)	
Week 4	1.55(1.33)	1.29(1.61)	0.512	0.89(1.04)	0.444
Week 8	1.47(1.32)	1.25(1.62)	0.140	-	-

For each variable, the values are expressed as the mean (SD).

*∗*Comparison based on the change of score.

## Data Availability

The data used to support the findings of this study are available from the corresponding author upon request.

## Supplementary Materials

Supplementary Materials Supplement 1. CONSORT 2010 Checklist. Supplement 2. STRICTOM checklist. Supplement 3. Calibration curves of references compounds determined. Supplement 4. HPLC chromatograms. Supplement 5. The name, source, and contents of references compounds of each ingredient
