# Supplementary material for: An External CAM Therapy (Tian Jiu) versus Placebo in Treatment of Allergic Rhinitis: A Pilot Single-Blinded, Three-Arm, Randomized Controlled Study
Source: Evid Based Complement Alternat Med. 2019 Apr 14;2019:6369754. doi: 10.1155/2019/6369754 (PMC6487095; doi:10.1155/2019/6369754)
Supplement: Supplementary Materials — Supplement 1. CONSORT 2010 Checklist. Supplement 2. STRICTOM checklist. Supplement 3. Calibration curves of references compounds determined. Supplement 4. HPLC chromatograms. Supplement 5. The name, source, and contents of references compounds of each ingredient [file 6369754.f1.doc]

Supplement 1 CONSORT 2010 checklist

| Section/Topic | Item No | Checklist item | Reported on page No |
| --- | --- | --- | --- |
| Title and abstract | | | |
|  | 1a | Identification as a randomised trial in the title | Page 1 |
|  | 1b | Structured summary of trial design, methods, results, and conclusions (for specific guidance see CONSORT for abstracts) | Page 3-4 |
| Introduction | | | |
| Background and objectives | 2a | Scientific background and explanation of rationale | Page 5-6 |
|  | 2b | Specific objectives or hypotheses | Page 6 |
| Methods | | | |
| Trial design | 3a | Description of trial design (such as parallel, factorial) including allocation ratio | Page 7 |
|  | 3b | Important changes to methods after trial commencement (such as eligibility criteria), with reasons | Not applicable |
| Participants | 4a | Eligibility criteria for participants | Page 8-9 |
|  | 4b | Settings and locations where the data were collected | Page 8 |
| Interventions | 5 | The interventions for each group with sufficient details to allow replication, including how and when they were actually administered | Page 9-12 |
| Outcomes | 6a | Completely defined pre-specified primary and secondary outcome measures, including how and when they were assessed | Page 13-14 |
|  | 6b | Any changes to trial outcomes after the trial commenced, with reasons | Not applicable |
| Sample size | 7a | How sample size was determined | Page 15 |
|  | 7b | When applicable, explanation of any interim analyses and stopping guidelines | Not applicable |
| Randomisation: |  |  |  |
| Sequence generation | 8a | Method used to generate the random allocation sequence | Page 14 |
|  | 8b | Type of randomisation; details of any restriction (such as blocking and block size) | Page 14 |
| Allocation concealment mechanism | 9 | Mechanism used to implement the random allocation sequence (such as sequentially numbered containers), describing any steps taken to conceal the sequence until interventions were assigned | Page 14 |
| Implementation | 10 | Who generated the random allocation sequence, who enrolled participants, and who assigned participants to interventions | Page 14 |
| Blinding | 11a | If done, who was blinded after assignment to interventions (for example, participants, care providers, those assessing outcomes) and how | Page 14 |
|  | 11b | If relevant, description of the similarity of interventions | Page 11 |
| Statistical methods | 12a | Statistical methods used to compare groups for primary and secondary outcomes | Page 15-16 |
|  | 12b | Methods for additional analyses, such as subgroup analyses and adjusted analyses | Not applicable |
| Results | | | |
| Participant flow (a diagram is strongly recommended) | 13a | For each group, the numbers of participants who were randomly assigned, received intended treatment, and were analysed for the primary outcome | Page 17-18  Figure 1 |
|  | 13b | For each group, losses and exclusions after randomisation, together with reasons | Figure 1 |
| Recruitment | 14a | Dates defining the periods of recruitment and follow-up | Page 17 |
|  | 14b | Why the trial ended or was stopped | Not applicable |
| Baseline data | 15 | A table showing baseline demographic and clinical characteristics for each group | Table 3 |
| Numbers analysed | 16 | For each group, number of participants (denominator) included in each analysis and whether the analysis was by original assigned groups | Figure 1 |
| Outcomes and estimation | 17a | For each primary and secondary outcome, results for each group, and the estimated effect size and its precision (such as 95% confidence interval) | Page 18-22  Figure 2, Table 3-4 |
|  | 17b | For binary outcomes, presentation of both absolute and relative effect sizes is recommended | Not applicable |
| Ancillary analyses | 18 | Results of any other analyses performed, including subgroup analyses and adjusted analyses, distinguishing pre-specified from exploratory | Not applicable |
| Harms | 19 | All important harms or unintended effects in each group (for specific guidance see CONSORT for harms) | Page 21-22 |
| Discussion | | | |
| Limitations | 20 | Trial limitations, addressing sources of potential bias, imprecision, and, if relevant, multiplicity of analyses | Page 25-26 |
| Generalisability | 21 | Generalisability (external validity, applicability) of the trial findings | Page 22-25 |
| Interpretation | 22 | Interpretation consistent with results, balancing benefits and harms, and considering other relevant evidence | Page 22-25 |
| Other information | | |  |
| Registration | 23 | Registration number and name of trial registry | Page 7 |
| Protocol | 24 | Where the full trial protocol can be accessed, if available | Page 7 |
| Funding | 25 | Sources of funding and other support (such as supply of drugs), role of funders | Page 7 |

**Supplement 2** Checklist for items in STRICTOM

| **Item** | **Detail** | **Page number** |
| --- | --- | --- |
| **1. Moxibustion rationale** | 1a) Type of moxibustion (e.g., direct moxibustion, indirect moxibustion, heat-sensitive moxibustion, moxa burner moxibustion, natural moxibustion) | Page 5-6 |
|  | 1b) Reasoning for treatment provided, based on historical context, literature sources, and/or consensus methods, with references where appropriate | Page 5-6 |
|  | 1c) Extent to which treatment was varied | Not applicable |
| **2. Details of moxibustion** | 2a) Materials used for moxibustion (e.g., moxa floss, moxa cone, moxa stick, herbal patches, and their sizes and manufacturers) | Page 9-11 |
|  | 2b) Names of acupoints (or location if no standard name) for moxibustion (uni/bilateral) | Page 11-12 |
|  | 2c) Number of moxibustion units and/or moxibustion time per point (mean and range where relevant) | Page 11-12 |
|  | 2d) Procedure and technique for moxibustion (e.g. direct/indirect, warming/sparrow-packing technique, warming needle, moxa box, heat-sensitive moxibustion) | Page 11-12 |
|  | 2e) Response sought (e.g., warm feeling, skin reddening, burning pain, heat-sensitization phenomenon) | Page 11-12 |
|  | 2f) Patient posture and treatment environment | Page 11 |
| **3. Treatment regimen** | 3) Number, frequency and duration of treatment sessions | Page 11-12 |
| **4. Other components of treatment** | 4a) Details of other interventions administered to the moxibustion group (e.g. acupuncture, cupping, herbs, exercises, lifestyle advice) | Not applicable |
|  | 4b) Setting and context of treatment protocol, and information and explanation to patients | Page 9 |
| **5. Treatment provider background** | 5) Description of treatment provider (qualification or professional affiliation, years in moxibustion practice and other relevant experience for professional, or any special training in advance for layman) | Page 12 |
| **6. Control and comparator interventions** | 6a) Rationale for the control or comparator in the context of the research question, with sources that justify this choice | Page 6 |
|  | 6b) Precise description of the control or comparator. If another form of moxibustion or moxibustion-like control is used, provide details as for Items 1 to 3 above. | Page 11 |
| **7. Precaution measures** | 7) Precise description of the precaution measures, if any | Page 11-12 |

**Supplement 3. Calibration curves of references compounds determined (n=3)**

| Compounds | Calibration curve | Correlation coefficient (r^2^) | Linear range (μg) |
| --- | --- | --- | --- |
| Euphadienol | y = 0.001x + 0.0016 | 0.9999 | 1.00~5.00 |
| Asarinin | y = 0.0008x + 0.068 | 0.9999 | 0.50~2.50 |
| Sinapine thiocyanate | y = 0.0012x - 0.7056 | 0.9998 | 1.60~8.00 |
| Tetrahydropalmatine | y = 0.0012x + 0.0583 | 0.999 | 0.25~3.00 |

**Supplement 4. HPLC chromatograms of *Euphorbia Kansui* T. N. Liou ex T.P. Wang (a) and the Euphadienol standard (b); Asarum sieboldii Miq (c) and Asarinin standard (d); Semen sinapis (e) and Sinapine thiocyanate standard (f); Corydalis yanhusuo W. T. Wang (g) and Tetrahydropalmatine standard (k).**


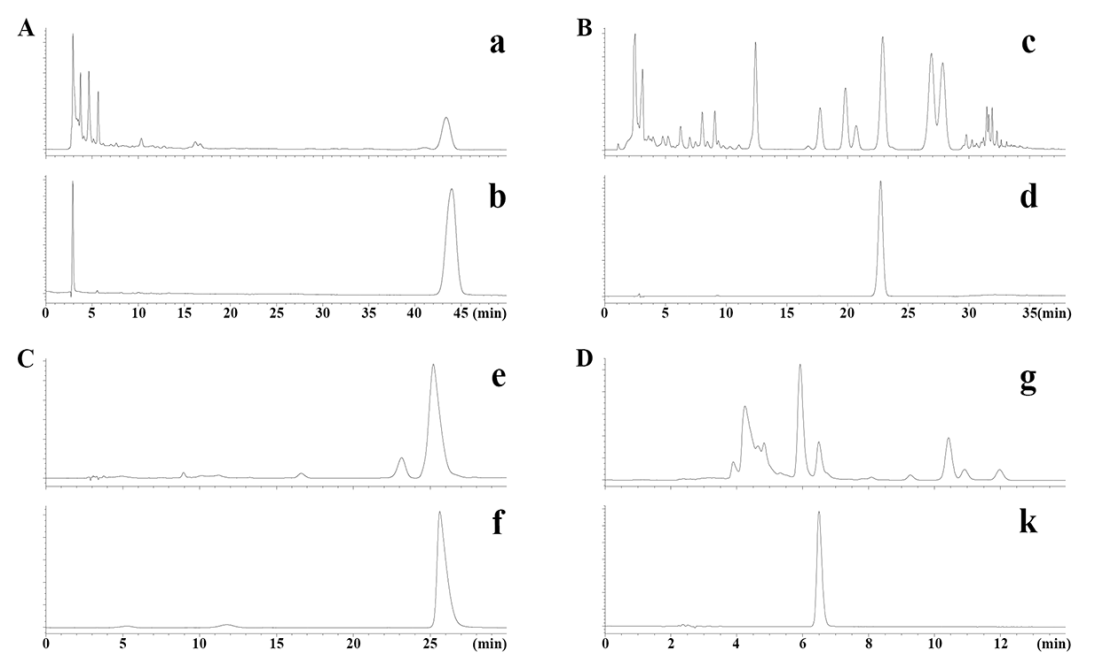


**Supplement 5 The name, source, contents of references compounds of each ingredient**

| **Ingredients** | | **Source** | **Medicinal parts** | **Methods / standards cited in the Chinese Pharmacopeia** | **Content (%)** |
| --- | --- | --- | --- | --- | --- |
| **Chinese name (*Pinyin*)** | **Latin name** |
| Zhi Gan Sui | *Kansui radix* | *Euphorbia Kansui* T. N. Liou ex T.P. Wang | Dry root | Contained not less than 0.12% of euphadienol | 0.12 |
| Xi Xin | *Asari radix et rhizoma* | *Asarum sieboldii* Miq. | Dry root and rhizome | Contained not less than 0.050% of asarinin | 0.230 |
| Bai Jie Zi | *Sinapis semen* | *Sinapis alba* L. | Dry seed | Contained not less than 0.50% of snapine thiocyanate | 0.65 |
| Yan Hu Suo | *Corydalis rhizoma* | *Corydalis yanhusuo* W. T. Wang | Dry tuber | Contained not less than 0.050% of tetrahydropalmatine | 0.570 |
